# Umbilical Cord Milking Improves Transition in Premature Infants at Birth

**DOI:** 10.1371/journal.pone.0094085

**Published:** 2014-04-07

**Authors:** Anup Katheria, Doug Blank, Wade Rich, Neil Finer

**Affiliations:** Division of Neonatology, UCSD Medical Center, University of California San Diego, San Diego, California, United States of America; University of Alabama at Birmingham, United States of America

## Abstract

**Background:**

Umbilical cord milking (UCM) improves blood pressure and urine output, and decreases the need for transfusions in comparison to immediate cord clamping (ICC). The immediate effect of UCM in the first few minutes of life and the impact on neonatal resuscitation has not been described.

**Methods:**

Women admitted to a tertiary care center and delivering before 32 weeks gestation were randomized to receive UCM or ICC. A blinded analysis of physiologic data collected on the newborns in the delivery room was performed using a data acquisition system. Heart rate (HR), SpO_2_, mean airway pressure (MAP), and FiO_2_ in the delivery room were compared between infants receiving UCM and infants with ICC.

**Results:**

41 of 60 neonates who were enrolled and randomized had data from analog tracings at birth. 20 of these infants received UCM and 21 had ICC. Infants receiving UCM had higher heart rates and higher SpO_2_ over the first 5 minutes of life, were exposed to less FiO_2_ over the first 10 minutes of life than infants with ICC.

**Conclusions:**

UCM when compared to ICC had decreased need for support immediately following delivery, and in situations where resuscitation interventions were needed immediately, UCM has the advantage of being completed in a very short time to improve stability following delivery.

**Trial Registration:**

ClinicalTrials.gov NCT01434732

## Introduction

Recently the 2012 ACOG guidelines recommended delayed cord clamping (DCC) for at least 30 seconds and up to one minute in preterm infants [1]. Despite the evidence and recommendations for DCC, there is still reluctance by the neonatal/obstetrical community to adopt this therapy because of possible conflict with immediate newborn resuscitation. Umbilical cord milking (UCM), in which the unclamped umbilical cord is immediately milked and clamped, results in rapid blood transfer from the placenta to the newborn allowing resuscitation of the premature infant to proceed without delay.

According to the Neonatal Resuscitation Program guidelines, heart rate is the most important indicator of infant well-being during neonatal resuscitation [2]. In 1962, Brady et al demonstrated that after early cord clamping there was a marked bradycardia in the term infants [3]. Dawson et al. described a median heart rate of <100 bpm at one minute of life in term and preterm neonates [4]. Bhatt et al demonstrated a 50% drop in pulmonary blood flow and an abrupt 40% drop in heart rate (due to cessation of umbilical venous flow from the placenta) in anesthetized fetal lambs receiving immediate cord clamping (ICC) [5].

To date there has not been a prospective randomized trial examining the effect of UCM on resuscitation interventions and the changes in heart rate and SpO_2_ immediately following delivery. UCM by may have a positive effect on heart rate and SpO_2_ in the premature infant immediately after birth, by maintaining pulmonary blood flow and reducing the amount of resuscitation needed. In 2010, prior to the new guidelines, immediate cord clamping was our standard of care. We hypothesized that umbilical cord milking would improve the transition of preterm infants at birth as demonstrated by higher heart rates and SpO_2_ during neonatal resuscitation. We also postulated that infants receiving UCM would require/receive less resuscitation determined by receipt of less supplemental FiO_2_ and ventilation, measured by averaged mean airway pressure.

## Methods

This was a randomized controlled trial conducted at the University of California, San Diego, a tertiary care facility with a 49 bed NICU. The protocol for this trial and CONSORT checklist are available as supporting information (see Checklist S1 and Protocol S1). Written informed consent was obtained from all participants (pregnant women <32 weeks who were admitted to the labor and delivery floor) in this study as approved by the University of California, San Diego Institutional Review Board. Dating was determined by the earliest ultrasound or last menstrual period.

Entry criteria included a gestational age of 23 0/7 to 31 6/7 and informed consent. Diamniotic dichorionic twins were eligible for enrollment. Each twin was treated as an individual subject and randomized separately. Exclusion criteria included monochorionic multiples, incarcerated mothers, placenta previa, concern or development of placental abruption, or refusal by the obstetrician. Infants were randomized and stratified by gestational age (23–28 6/7 or 29–31 6/7) to ensure equal numbers of neonates <29 weeks in each arm.

Immediately prior to delivery, the infants were randomized to UCM or ICC and the obstetricians were made aware of the treatment. The neonatal fellow attending the delivery recorded the elapsed time from when the infant was delivered until the umbilical cord was clamped by the obstetrician. The obstetrician performed UCM prior to clamping of the umbilical cord. The obstetrician held the infant below the mother's introitus after a vaginal delivery or below the level of the incision at a cesarean section. A second obstetrician milked approximately 20 cm of umbilical cord toward the baby over two seconds, repeating this process a total of three times prior to clamping the cord and previously described [6],[7].

Relevant prenatal and neonatal data were obtained from the medical record. Maternal age, placental complications, antenatal steroid and magnesium administration, and mode of delivery were obtained from the medical records. Infant data, including gestational age, gender, weight, placental weight, Apgar scores at 1 and 5 minutes, and the need for intubation or chest compressions in the delivery room (DR), were also obtained from the medical records.

At UCSD Medical Center, newly born premature babies are evaluated in a designated resuscitation room. outfitted with additional monitoring equipment including ECG electrodes, pulse oximetry, a transducer to detect airway pressure, and data acquisition system that converts the analog data to digital so that it may be recorded in a tracing and analyzed. Real-time physiologic data was captured using our previously described data acquisition system [8]. The standard data captured includes pulse rate and oxygen saturation values from an oximeter, airway pressure, FiO_2_, and heart rate from ECG.

Immediately after placing the infant on the radiant warmer, ECG electrodes were placed to evaluate heart rate. ECG can pick up a continuous HR within 2 seconds of application, which is significantly faster than with a pulse oximeter (PO) [8],[9]. ECG readings were obtained from defibrillator analog output of a HR monitor (M3046A Phillips Medical Systems, Andover, MA). The HR produced from the ECG tracing was considered reliable if the QRS waveform was visually identifiable on the tracing during data analysis. If the QRS waveform was not clear on the tracing, HR was obtained from the PO tracing if available.

A pulse oximeter sensor was applied to the baby's right palm or wrist immediately after the baby was placed on the warming bed, then immediately connected to the oximeter. A Radical 7 (Masimo, Irvine CA) oximeter was used to determine SpO_2_ and pulse rate if ECG was not reliably detecting heart rate. The analog output was 1 sample per second and was set to a 2 second averaging interval and maximum sensitivity. Data were considered reliable when the tracing showed a steady pulse rate signal. The time to obtain a reliable HR via ECG and PO was recorded.

Preterm newborns who required resuscitation received initial mask positive pressure using a t-piece resuscitator (Neopuff, Fisher & Paykel, Auckland, NZ), with a starting peak inspiratory pressure(PIP) of 30 cmH_2_O and an end expiratory pressure (PEEP) of 5 cmH_2_O. The data acquisition system recorded the pressure of each manual inflation delivered. All physiologic data captured from the monitors was converted to digital form using a data acquisition system.

Using the data acquisition system, HR, SpO_2_, and FiO_2_ were recorded at single time points every 30 seconds from the time the baby was placed on the warming bed until ten minutes. A 10 second rolling average of mean airway pressure in cmH_2_0 (MAP) was collected every 30 seconds from the time the baby was placed on the warming bed until ten minutes. Maximum FiO_2_ and maximum peak inflation pressures (PIP) in cm H_2_0 were also recorded. If the tracings of the infants were not available, the maximum FiO_2_ and maximum PIP received while in the DR was obtained from the resuscitation documentation infant's medical record.

Data from the oximeter and an AX300 oxygen analyzer (Teledyne Analytical Instruments, City of Industry, CA) were acquired at 1 sample/sec and processed by a 4 channel 12 bit A/D board (DI-158U, Dataq Instruments, Akron, Ohio) connected via a USB port to a netbook computer, (Dell, Inc. Round Rock, Tx.). The saturation of the infant is plotted against the high (50^th^%) and low (10%) target ranges and displayed to the resuscitation team on a video screen. The SpO_2_ ranges used are drawn from data for infants of 32–36 weeks gestation who did not require any intervention including provision of supplemental oxygen [10]. The 10^th^ and 50^th^ percentiles have been used as the upper and lower saturation targets [9], [11].

Serial changes in HR, SpO_2_, FiO_2_, and MAP over the first minutes of life were compared in infants in each intervention arm using repeated analysis of variance (ANOVA). Demographic data are presented as numbers and proportions for categorical variables or means with SD for normally distributed continuous variables and medians with when the distribution were skewed. Normally distributed continuous outcome variables were analyzed with independent samples T-Test and nonparametric continuous outcome variables were analyzed with the Mann-Whitney U test. Fishers exact test and Chi Square test were used to analyze categorical outcome variables. Significance was set at a p value less than 0.05. We performed descriptive statistics using IBM SPSS Statistics 21.0 (SPSS, Inc, Chicago, IL).

This study was a part of a larger study designed to detect a difference in superior vena cava flow using cardiac ultrasound at 6 hours between neonates treated with UCM compared with ICC [12]. A sample size calculation using retrospective data with a two-sided alpha of 0.05 and 80% power determined that 60 subjects were needed, 30 in each arm, to detect a 25% difference in superior vena cava flow.

## Results

One hundred nineteen women were approached for consent between February 1, 2011 and January 31, 2013. Twenty-four declined to participate. Thirty women carried past 32 weeks and were not eligible for participation. Five infants (3 assigned to the UCM group and 2 assigned to the ICC group) were excluded after being randomized because of pre-defined criteria. These criteria included acute abruption (N = 2), cutting through the placenta (N = 2) or deemed too unstable (N = 1). Two mothers gave consent but their infants were excluded because the team was unaware they were eligible for the study at the time of delivery. The remaining 58 women (two sets of twins, N = 60 neonates) met inclusion criteria for umbilical cord milking or immediate cord clamping.

**Figure 1:**
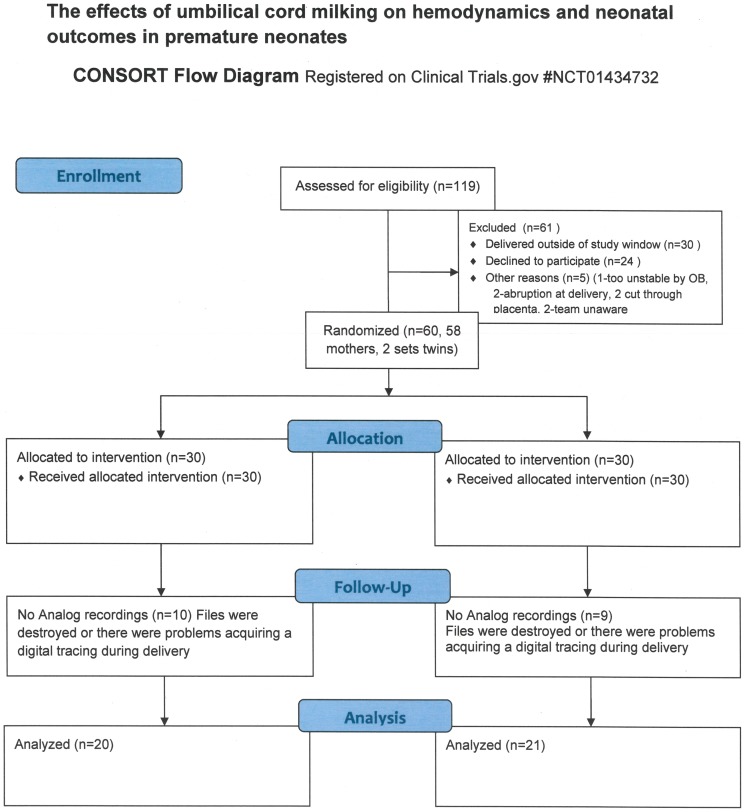
Consort Diagram.

All 60 infants had documented resuscitation information. There were no differences in the time to clamp the umbilical cord, 1 and 5 minute Apgar scores, the need for continuous positive airway pressure (CPAP), positive pressure ventilation (PPV), intubation, chest compressions, or the administration of epinephrine, Nineteen did not have information available from the data acquisition system during resuscitation (10 from the UCM group and 9 from the ICC group). Reasons included files being destroyed (this data collection is generally done as a quality project, and files that will not be reviewed are erased immediately), or there were problems acquiring a digital tracing during delivery. Of the 60 enrolled patients, 41 infants, 20 assigned to the UCM group and 21 assigned to the ICC group, had digital tracings of heart rate, SpO_2_, FiO_2_ and mean airway pressure from the DR (Figure 1). There were no differences in gestational age at birth, birth weight, gender, placenta weight, exposure to antenatal steroids or antenatal magnesium, mode of delivery between the groups, or time to clamping of the umbilical cord between the two groups of infants with digital tracings (Table 1). Infants who received ICC required higher peak FiO2 and peak inspiratory pressures compared to those receiving UCM.

**Table 1:** Demographics and results: Umbilical Cord Milking vs. Immediate Cord Clamping, N = 41.

	UCM (N = 20)	ICC (N = 21)	p value
Gestation, weeks, median (IQR)	29 (27–30)	27 (26–31)	0.26
Birth weight, grams, mean (SD)	1168±414	992±324	0.13
Male (%)	13 (65%)	13 (62%)	P = 0.8
Placenta weight, grams, mean (SD)	273±106	273±93	P = 0.7
Antenatal steroids (%)	20 (100%)	21 (100%)	1
Antenatal magnesium (%)	17 (85%)	18 (86%)	1
Vaginal delivery (%)	6 (30%)	5 (24%)	0.7
Time to cord clamping, seconds, median (IQR)	14 (10–16)	11 (10–15)	0.57
Maximum FiO2, median (IQR)	40 (21–46)	50 (40–65)	0.03
Maximum PIP, cm H20, median (IQR)	30 (30–40)	40 (30–40)	0.02
Apgar 1 minute, median (IQR)	5 (3–5)	6 (4–7)	0.16
Apgar 5 minute, median (IQR)	6 (4–7)	8 (7–9)	0.13

**Figure 2:**
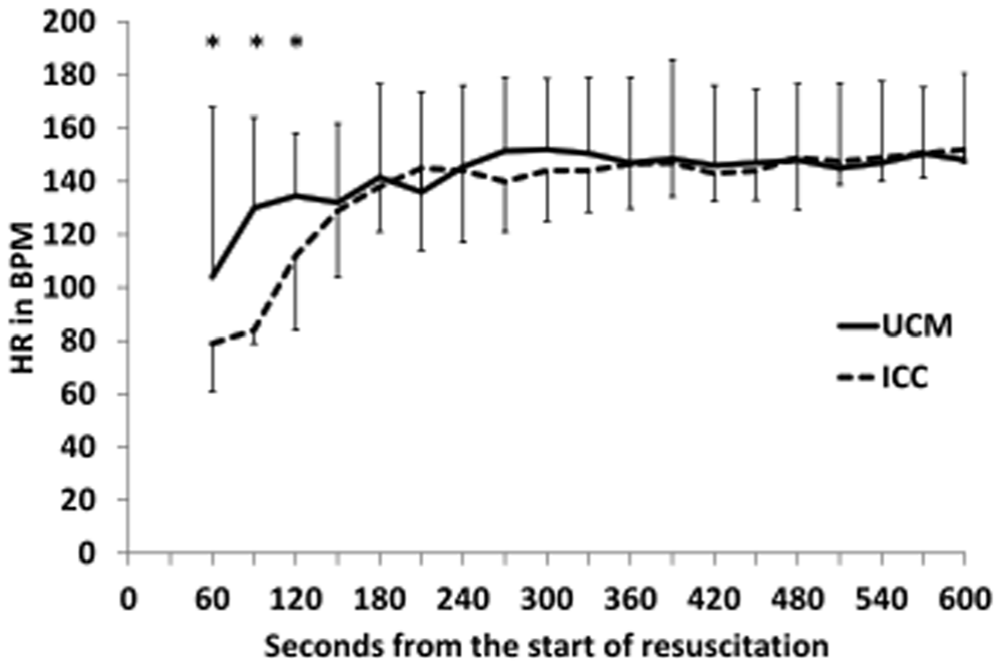
Changes in Heart Rate over time in infants receiving umbilical cord milking (UCM) compared to immediate cord clamping (ICC). The babies receiving UCM had significantly higher heart rates over the first 5 minutes of resuscitation (ANOVA *P* = 0.032). *P<0.05 compared to ICC.

**Figure 3:**
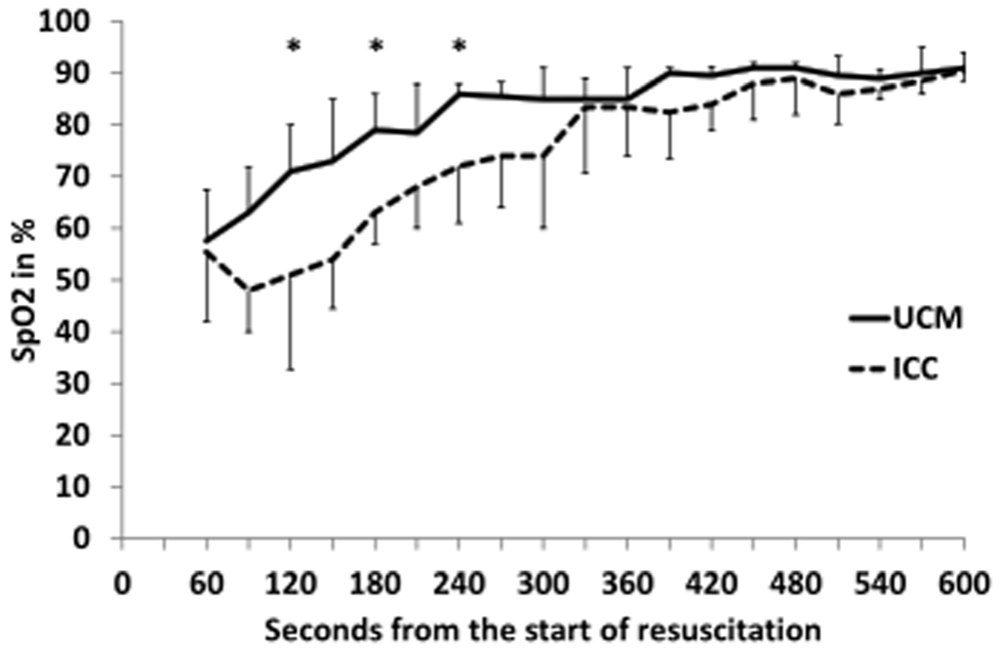
Changes in Oxygen Saturation (SpO_2_) over time in infants receiving umbilical cord milking (UCM) compared to immediate cord clamping (ICC). The babies receiving UCM had significantly higher SpO2 over the first 5 minutes of resuscitation (ANOVA *P* = 0.015). *P<0.05 compared to ICC.

**Figure 4:**
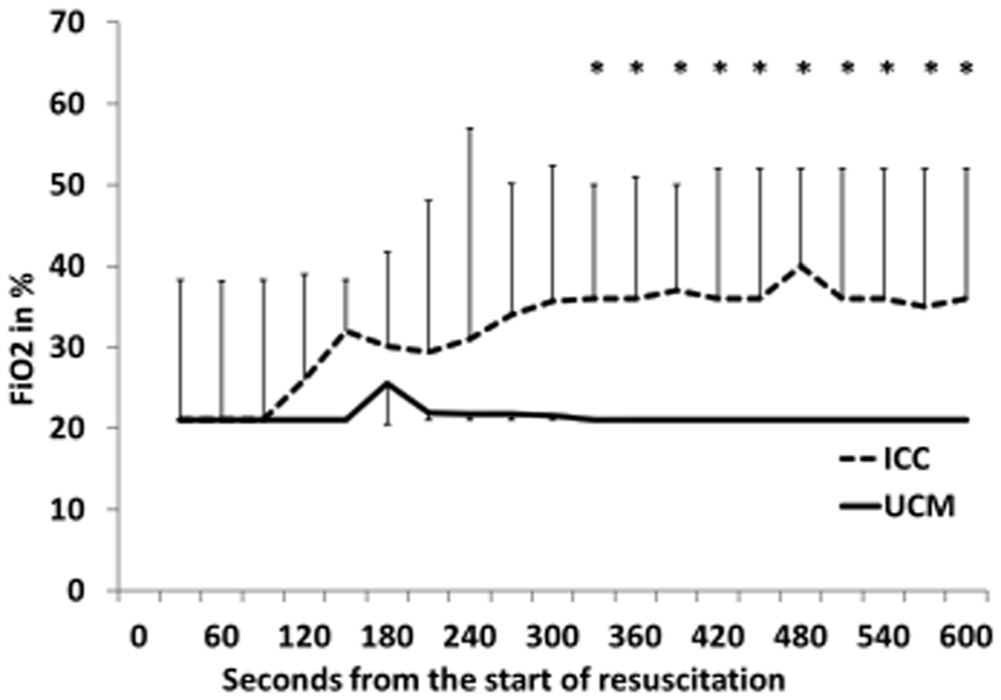
Changes in Inspired Fractional oxygen concentration (FiO_2_) over time in infants receiving umbilical cord milking (UCM) compared to im mediate cord clamping (ICC). The babies receiving UCM received significantly less supplemental FiO2 over the first 10 minutes of resuscitation (ANOVA, *P* = 0.04). *P<0.05 compared to ICC.

**Figure 5:**
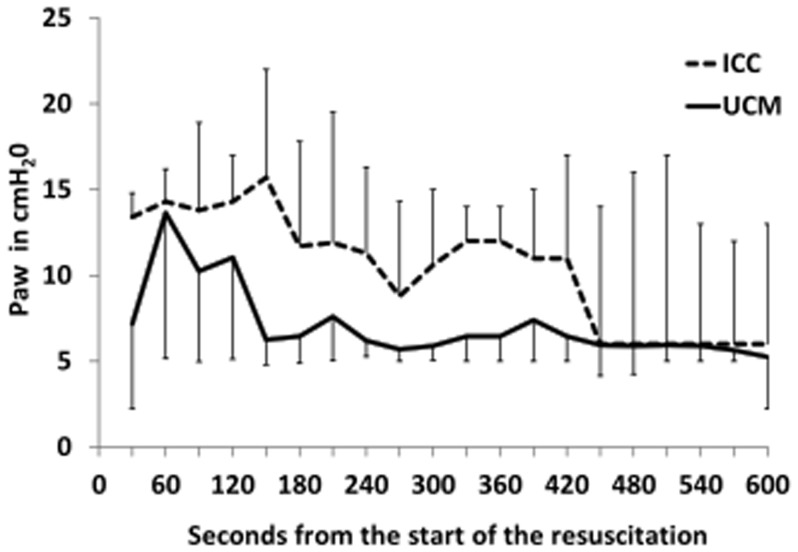
Changes in mean airway pressure (MAP) over time in infants receiving umbilical cord milking (UCM) compared to immediate cord clamping (ICC). During the first five minutes of resuscitation, there was a non-significant trend towards a lower mean airway pressure requirement in infants in the UCM (ANOVA, *P* = 0.13).

Neonates who received UCM and had digital tracings, had significantly higher heart rates using over the first 5 minutes of resuscitation (p = 0.03, Figure 2), with median heart rates higher at 60, 90, and 120 seconds (p<0.05, Figure 2). UCM infants also had significantly higher SpO_2_ during the first 5 minutes of resuscitation (p = 0.015, Figure 3), with median SpO_2_ higher at 120, 180, and 240 seconds (p<0.05, Figure 3). Neonates who received UCM required significantly less FiO_2_ during the first 10 minutes of resuscitation (p = 0.04, Figure 4). A greater number of infants receiving umbilical cord milking never required supplemental oxygen in the delivery room (40 vs. 5 percent, p = 0.009). For infants <26 weeks gestation 10/10 infants received oxygen in the ICC group and 3/5 infants received oxygen in the UCM group. There was a non-significant trend towards a lower mean airway pressure requirement in infants in the UCM (p = 0.13, Figure 5).

## Discussion

This is the first randomized controlled trial to demonstrate that premature infants receiving UCM had higher HR and SpO_2_, and received less FiO_2_ during resuscitation than infants receiving ICC. The benefits of UCM on HR and SpO_2_ appear to be most significant in the first few minutes of life. In an animal model using preterm lambs, Bhatt et al. demonstrated that ICC leads to bradycardia until ventilation was established, but lambs that had delayed cord clamping (DCC) did not have bradycardia. In addition, lambs with ICC had a rapid rise in carotid artery pressure, carotid arterial blood flow, and pulmonary blood pressure starting at 4 beats after clamping and continuing for 30 seconds, which was followed by a decrease in to below baseline by 90 seconds after clamping the cord. The lambs with DCC until ventilation was established did not have significant changes in carotid artery pressure, carotid artery blood flow, or pulmonary artery pressure after clamping the umbilical cord [5]. UCM offers an alternative to DCC because UCM does not delay resuscitation. In our study, the infants receiving UCM had median HR>100 BPM at 60 seconds and a gentle rise in both HR and SpO_2_ after birth. A study to determine if UCM prevents bradycardia and swings in carotid artery pressure, blood flow, and pulmonary artery pressure after cord clamping is needed.

Placental transfusion has shown to be beneficial to the preterm infant. A recent Cochrane Review demonstrated that delaying umbilical cord clamping for at least 30 to 120 seconds in preterm infants decreased the need for red blood cell transfusion, and intraventricular hemorrhage. However placental transfusion had no effect on the APGAR scores at 1, 5, and 10 minutes [13]. Kaempf et al. report that DCC in premature infants <1500 g had higher 1 minute APGAR scores, less need for supplemental oxygen and less bag and mask ventilation and concluded that delayed cord clamping is safe in singleton premature infants [14]. Using a data acquisition system, we have been able to expand on the findings of the previous study to show that UCM improves neonatal transition compared with ICC.

Nineteen infants had missing data from the DR, however these infants were matched between the UCM and ICC groups and did not have any differences in antenatal outcomes. Our study also required antenatal consent and therefore all of the infants were exposed to antenatal steroids and the majority received magnesium sulfate. We now have compelling evidence that trials which require consent before delivery are likely to enroll a population that is not as sick as those eligible but not enrolled, and decreases the generalizability of this large trial. Rich et al found that infants who were approached for participation in the SUPPORT trial were significantly different from non-approached infants who were eligible for this trial and were born in the recruiting centers. Enrolled infants were more likely to be white, have mothers with better education and health insurance, and have prenatal care [15]. They were less likely to be in the most immature gestational age week for this study. Of greater concern is that the enrolled infants were significantly more likely to have received a full or partial course of antenatal steroids (ANS) with only 3.4% of such infants receiving no ANS compared with 15.7% for the infants who were not enrolled. This study also had a small number of infants <29 weeks GA (n = 23) [15]. A pilot study of 75 extremely premature neonates <29 weeks randomized to UCM or ICC demonstrated a 50% reduction in total IVH [7]. It is possible that infants <29 weeks may have greater benefits at delivery with UCM.

We could not for practical purposes have a blinded intervention at the time of delivery since the neonatal fellow receiving the infant also led the resuscitation. However, our primary outcome for the trial was not the delivery room changes, and these data were collected for a secondary analysis. In addition, there was no difference in the mean airway pressure despite the changes demonstrated in heart rate and SpO2. One could assume that in order to achieve higher heart rates and oxygen saturations in the first few minutes that the UCM group should have received higher mean airway pressure, but this was not the case.

When our trial began in 2011, our standard practice was to begin resuscitation with an inspired fractional oxygen concentration of (FiO_2_) of 0.4 (n = 11 infants in our dataset). However by the middle of 2010 we changed an initial FiO_2_ of 0.21. Since our study was block stratified in groups of 10 we were able to ensure that there were similar numbers of infants who started at 0.21 compared to 0.4. There was no difference in the heart rate, spO_2_, or calculated mean airway pressure between those infants started on 0.4 or 0.21 (data not shown).

Our data would support the concept that the volume of blood given infants through UCM or DCC improves pulmonary blood flow and assists lung expansion. In 1958 Jaykka, demonstrated that the alveolar patency occurs in response to the filling of the surrounding capillaries.^12^ Since HR and SpO_2_ increase as a direct result of pulmonary expansion, this may explain why the infants receiving UCM had higher HR and SpO_2_ in the first minutes of resuscitation. UCM may prevent lung injury in the premature infant at birth by improving lung expansion and decreasing the need for mechanical ventilation and the associated baro/volutrauma. Prevention of lung injury at birth may explain the findings of decreased need for oxygen at 36 weeks in Hosono's trial of infants <28 weeks [6] and in our trial [12]. Our study was powered to detect a difference in superior vena cava flow after birth, and was not powered to evaluate the physiologic responses following delivery [12]. Currently we are conducting a larger trial with a waiver of antenatal consent (n = 600, NCT01866982) comparing delayed cord clamping to cord milking which will be collecting similar data which is needed to confirm our results.

## Conclusions

Premature infants who receive UCM have higher heart rates and SpO_2_ and require lower amounts of oxygen after delivery when compared to infants whose umbilical cords are clamped immediately after birth. Premature infants benefit from a placental transfusion as seen in the delivery room and UCM offers an approach that can be used in the most compromised infants. Further studies are required that are adequately powered to evaluate both short and longer term outcomes in premature infants receiving umbilical cord milking.

## Supporting Information

Protocol S1 **Research Plan as approved by the University of California at San Diego.**
(DOC)

Checklist S1 **CONSORT Checklist for Randomized Controlled Trials.**
(DOCX)
