# Supplementary material for: Umbilical Cord Milking Improves Transition in Premature Infants at Birth
Source: PLoS One. 2014 Apr 7;9(4):e94085. doi: 10.1371/journal.pone.0094085 (PMC3978008; doi:10.1371/journal.pone.0094085)
Supplement: Protocol S1 — Research Plan as approved by the University of California at San Diego. (DOC) [file pone.0094085.s001.doc]

| University of California, San Diego (UCSD) and Rady Children’s Hospital – San Diego (RCHSD) **IRB Protocol Application**  **RESEARCH PLAN INSTRUCTIONS**  These are instructions for filling out the Research Plan that is available in MS Word format from the IRB website: [https://irb.ucsd.edu](https://irb.ucsd.edu/)  The headings on this set of instructions correspond to the headings of the Research Plan.  General Instructions: Enter a response in for all topic headings.  Enter “Not Applicable” rather than leaving sections blank if the section does not apply to this project.  Version date:10/09 |
| --- |
| 1. **PROJECT TITLE:** |
| A randomized controlled trial of umbilical cord milking versus immediate cord clamping on systemic blood flow in premature infants. |
| **2. PRINCIPAL INVESTIGATOR:** |
| Anup Katheria, M.D. |
| **3. FACILITIES:** |
| UCSD Medical Center, Infant Special Care Center |
| **4. ESTIMATED DURATION OF THE STUDY:** |
| The duration of this study will include recruitment of study subjects over one year, analysis of the data over six months after all studies have been completed, and manuscript preparation over 18 months. Total study time will be three years. |
| **5.** **SPECIFIC AIMS:** |
| To determine whether milking of the umbilical cord at delivery produces a greater improvement in systemic blood flow as measured by superior vena cava (SVC) flow when compared with immediate clamping of the umbilical cord. |
| **6. BACKGROUND AND SIGNIFICANCE:** |
| Intraventicular hemorrhage (IVH) is one of the most devastating complications in the neonatal period. Neurodevelopmental impairment, which occurs more commonly after IVH, is a significant morbidity in the premature infant population. It has been suggested that impaired cardiovascular hemodynamics may be contributory to the development of IVH. One measure of cerebral hemodynamic function is the sonographic estimate of blood flow in the Superior Vena Cava. Investigators have demonstrated the association of low Superior Vena Cava (SVC) flow and the subsequent development of IVH. Low SVC flow has been shown to be more sensitive in predicting late onset IVH than hypotension (Kluckow, 2000). SVC flow provides an estimate of upper body cardiac input and is not affected by a persistant ductus arteriosus (PDA) or a patent foramen ovale (PFO) shunts making it a more useful measure of systemic blood flow than traditional measures of cardiac output such as left ventricular output. It has also been shown to be a useful predictor of impaired neurologic outcome in preterm infants whether or not IVH is present (Hunt, 2004).  Unfortunately, no medical intervention to date whether it be inotropic medication or afterload reduction has been shown to prevent low SVC flow (Osborn, 2002). However, in a recent meta-analysis of 10 small trials, delayed cord clamping (DCC) has been shown to reduce the incidence of IVH without any adverse effects (Rabe, 2007). There is currently no published data describing the changes in SVC flow with DCC. Despite this evidence for benefit and safety there is still reluctance by the neonatal/obstetrical community to adopt this therapy because it may conflict with the perceived need for immediate resuscitation. An alternative to DCC is umbilical cord milking or stripping (UCM) in which the unclamped umbilical cord is milked 2 to 3 times before it is clamped in order to auto-infuse blood into the preterm neonate. In a recent study by Hosono et al (2008) about 20 cm of umbilical cord was milked over 2 seconds and was repeated one to two times. They demonstrated an increase in the mean hemoglobin, blood pressure and urine output and a reduction in the need for blood transfusions and days to regain birthweight. A recently published study (Rabe, 2011) compared UCM with DCC in infants <33 weeks gestational age. While no major clincal outcomes were found between UCM and delayed cord clamping, infants who had UCM had higher Hb at 42 days of life suggesting an ability to maintain blood volume or better erythropoiesis. Studies dating back to 1954 (McCausland et al) suggested that UCM led to a more sustained rise in RBC count than delayed cord clamping in term infants (delayed for 5 minutes).  Umbilical cord milking occurs more rapidly than delayed cord clamping and therefore allows prompt resuscitation and warming of the premature newborn. One important risk factor for IVH is thought to be rapid volume overload (Goldberg, 1980). However, in both the Rabe and Hosono studies there was a non-significant *decrease* in IVH in the UCM group. These studies were not adequately powered to find differences in IVH. The effects of UCM on systemic blood flow have not been evaluated. No study to date has evaluated the transitional hemodynamics using echocardiography after UCM in premature infants. The findings of both studies, which have evaluated UCM in preterm infants, are outlined below. Our study will include infants <32 weeks similar to both the Rabe and Hosono study. The potential for reduction of morbidities such as IVH will be more pronounced in more immature infants since the incidence of IVH is much greater in that population. In addition because cord milking occurs over a shorter period of time, it is ideal for a more immature population allowing any necessary newborn resuscitation interventions to begin quickly.   | Study | Hosono, 2008, 2009 | | | Rabe, 2011 | | | | --- | --- | --- | --- | --- | --- | --- | | Enrollment Criteria, GA | <29 weeks | | | 24 0/7 – 32 6/7 weeks | | | | N=# infants per group | ICC  N=20 | UCM  N=20 | P | DCC  N=31 | UCM  N=27 | P | | Birth weight (g) | 846 ± 171 | 836 ± 223 | .43 | 1,263±428 | 1,235±468 | .81 | |  |  |  |  |  |  |  | | Gestational age (wk) | 26.6±1.3 | 27 ±1.5 | .14 | 29.2±2.3 | 29.5±2.7 | .61 | | 24–27 wk | not distinguished | not distinguished |  | 10 | 7 | .59 | | 28–32 wk | 21 | 20 | .59 | | Male, female | 13,7 | 10,10 | .34 | 17, 14 | 11, 16 | .31 | | # ROP | 10 | 6 | .14 | 0 | 1 | .28 | | # needing Transfusion | 14 | 7 | .03 | 15 | 17 | .40 | | All IVH | 5 | 3 | .43 | 7 | 3 | .31 | | Initial Hemoglobin (g/dL) | 14.1 | 16.5 | <.01 | 17.3 | 17.5 | .71 | | # Died | 3 | 2 | .63 | 4 | 2 | .67 | |
| **7. PROGRESS REPORT/PRELIMINARY STUDIES:** |
| This is the first study of its kind done by our group. |
| **8. RESEARCH DESIGN AND METHODS: (guideline length is 3-6 pages)** |
| **Study Design:** The proposed study will be a single blinded randomized controlled trial designed to evaluate cardiovascular changes during fetal to neonatal transition in premature infants (<32 weeks) receiving umbilical cord milking (UCM) at birth compared to standard care infants having immediate cord clamping (ICC). The investigators performing the echocardiograms will be blinded to the allocation of the intervention. The primary outcome will be the difference in SVC flow between the UCM and the ICC group. We postulate that infants in the UCM group will have increased SVC flow compared to gestational age-matched controls in the ICC group at each time point.  Secondary hypotheses include:   1. Infants randomized to UCM or ICC will have no difference in the use of delivery room interventions. 2. Infants receiving UCM will have an increased serum hemoglobin at 12 hours of life, higher blood pressures in the first 30 hours of life, right ventricular output, urine output, and the number of days at which first blood transfusion occurs compared to ICC. At the time of blood transfusion the hemoglobin, respiratory support including inspired fraction of oxygen (FiO2), heart rate, blood pressure, and the clinical indication will be recorded. 3. Infants receiving UCM will have decreases in serum lactate levels, lower pulmonary artery pressure, less right to left shunting at the level of the PDA and PFO, less need for cardiovascular support, less IVH, less neurodevelopmental impairment at 18 to 36 month, and fewer blood transfusions compared to ICC. 4. Infants receiving UCM or ICC will have no differences in Apgar scores, cord gases, admission temperature, intraventricular hemorrhage, total serum bilirubin or duration of phototherapy.   **Design Methodology**  **Intervention:**  After informed consent is obtained from the parents of infants with gestational age < 32 weeks (by best obstetric dating), preterm neonates will be randomly assigned by sealed envelopes opened immediately before delivery to receive either standard care with immediate clamping of the umbilical cord (ICC) or umbilical cord milking (UCM). We will use a block randomization scheme by a gestational age cutoff of 28 weeks to ensure than an equal number of infants <28 weeks are in each arm. Multiples (twins or triplets) will also be individually randomized. The obstetricians will be made aware of the type of intervention prior to delivery of the infant. A member of the research team will record the time elapsed from when the subjects buttocks are delivered from the vagina or uterus (or head if in breech position) until the time the umbilical cord is clamped by the obstetrician in both arms of the study. UCM will be performed by the obstetric team by having the delivering obstetrician hold the infant below the mother’s introitus at vaginal delivery or below the level of the incision at cesarean section and having the assistant (the second obstetrician) milk about 20 cm of umbilical cord over 2 seconds and repeating two additional times as described previously (Hosono, 2006). This should take about 10 seconds to complete before the obstetrician clamps the umbilical cord. The number of times the cord is milked in the UCM group will be recorded. The assigned intervention can be aborted if the delivering obstetrician or attending neonatology team determines that immediate resuscitation is necessary.  **Cardiac Doppler Ultrasounds:**  The investigators performing the echocardiograms will be blinded to which intervention the patient received. Initial echocardiograms will be performed in infants enrolled in the study as soon as possible after birth within the first 6 hours of life. Serial echocardiograms will then be performed at approximately 18, and 30 hours of life. There will be a margin of plus or minus 6 hours at each time point to ensure that the echocardiographer has adequate availability to perform the studies.  If any structural abnormalities are found including a ventricular septal defect, the attending pediatrician will be notified of the abnormal echocardiogram. The consent will clearly state that these echos are not for diagnostic purposes. Any additional studies including an official complete echocardiogram and or cardiology consultation will be left to the discretion of the attending pediatrician, as he/she deems necessary.  ***Superior vena cava (SVC) flow***  SVC flow will be performed by imaging the superior vena cava from the parasternal long axis window in a sagittal plane to obtain the vessel diameter and from the subcostal window to obtain the velocity. The internal diameter of the vessel will be measured by obtaining an M-mode tracing of the vessel at the junction of the SVC and the right atrium. The maximum and minimum diameter will be measured for three cardiac cycles and all 6 measurements averaged. The velocity time integral will be obtained from the subcostal view with the transducer placed as near the umbilicus as possible to minimize the angle of insonation. The pulse wave Doppler range gate will be placed at the junction of the SVC and the right atrium. The complete velocity time integral from 10 consecutive cardiac cycles displaying laminar flow will be obtained and averaged. SVC flow will be calculated by measuring the average velocity time integral and multiplying it by the average cross sectional area of the superior vena cava (mm), and the heart rate (beats per minute).  ***Right Ventricular Outflow (RVO)***  RVO will be performed by imaging the pulmonary artery from the parasternal long axis window in the sagittal plane to obtain the vessel diameter and the velocity. The complete velocity time integral from 5 consecutive cardiac cycles displaying laminar flow will be obtained and averaged. RVO will be calculated by measuring the velocity time integral and multiplying it by the cross sectional area of the pulmonary artery (cm), and the heart rate (beats per minute).  ***Left Ventricular Outflow (LVO)***  LVO will be performed by imaging the aorta from the parasternal long axis window in the sagittal plan to obtain the vessel diameter and from the apical window to obtain the velocity. The complete velocity time integral from 5 consecutive cardiac cycles displaying laminar flow will be obtained and averaged. LVO will be calculated by measuring the velocity time integral and multiplying it by the cross sectional area of the aorta (cm), and the heart rate (beats per minute).  All flow measures (SVC flow, RVO, and LVO) will be indexed to the subject’s weight in kg.  ***Ductus arteriosus***  The ductus will be imaged from the high parasternal window with the transducer angled to obtain a view of the ductus throughout its course from the pulmonary artery to the descending aorta. A color Doppler clip will be stored and the ductus diameter measured at the point of maximal constriction by frame-by-frame analysis of the images. Three measurements will be taken and averaged. The direction of flow will be assessed by placing the pulse wave Doppler range gate in the center of the ductus and recording the flow pattern. The direction of flow will be categorized as all left to right, all right to left, or bidirectional. If bidirectional, the percentage of right to left flow will be calculated by measurement of the time of right to left component over the time of the entire cardiac cycle.  ***Atrial Shunt***  Atrial shunting was imaged from the subcostal window and determined by measuring the diameter of the color flow jet across the atria. The color scale was lowered until atrial flow was seen and the gain was increased until the color signal was optimized. The pulsed wave Doppler range gate was placed in the foramen ovale and the pattern of flow was classified as left to right or bidirectional.  ***Measurement of Pulmonary Artery Pressure***  When Tricuspid regurgitation is detected with color Doppler flow imaging, the peak jet velocity of the pan-systolic regurgitant flow will be determined by pulse-wave Doppler method from the apical four-chamber view and converted to a pressure fall between the right ventricle and atrium by application of the modified Bernoulli equation P = 4V^2^, where P is the pressure fall in mmHg and V is the maximal regurgitant flow velocity in meters per second. This method has been validated in infants (Hunter, 1992).  DATA COLLECTION: In addition to the echocardiogram measurements, demographic, baseline, and outcome data will be collected from the mother’s and infant’s medical record (including blood gas, respiratory care records, and radiology records).  Relevant prenatal and neonatal information will be obtained from the chart including:   - Maternal Age - Maternal parity - Reason for delivery - Maternal medical history and complications during labor (Chorioamnionitis, Pre-eclampsia, Diabetes) - Medications during labor/delivery - Race/Ethnicity - Mode of delivery (Cesarean Section vs Vaginal Delivery), spontaneous or assisted. - Birthweight - Gestational Age (by best obstetric dating) - Birth weight percentile - Gender - Apgar Scores (1min/5min) - Cord Gases (if available) - DR interventions: oxygen (highest FiO2), CPAP, PPV (mask, ETT), Intubation, Surfactant, Chest compressions, epinephrine - Admission Temperature   The following neonatal variables will be recorded for assessment of secondary outcomes over the total hospitalization unless otherwise specified:   - Initial hemoglobin value at birth (done routinely for clinical use) and at 12 hours of life obtained from a indwelling line or from a venipuncture only if not already done for clinical purposes. - Hemoglobin Value at birth and at 12 hours of life from the pulse oximter. - Presence of polycythemia (defined as a central Hct >65 g/dL) - Age of first transfusion (days) - Peak serum bilirubin - Duration of phototherapy (days) - Duration of mechanical ventilation, positive pressure, and oxygen supplementation - Blood pressure, heart rate at 6, 12, 18, 24, and 30 hours of life - Cardiac Output and Stroke Volume from Electric Cardiometry (Oksypa Medical, Germany). - Urine output ml/kg/day over the first 72 hours - Cardiovascular support (use of volume boluses, steroids, and pressors) - Head ultrasound findings (from report of routine studies performed for clinical care/screening) including presence of periventricular leukomalacia (PVL) - MRI findings (if available from routine studies done for clinical care)   **ULTRASOUND OPERATORS**  The primary ultrasound operators will be the principal investigator (Anup Katheria) and co-investigator (Tina Leone). Drs. Katheria and Leone have had extensive bedside ultrasound training and experience in functional echocardiography. In addition they have participated in two other functional echocardiography trials. There will be some discrepancy between the two operators however as in their previous studies the images will be analyzed together to minimize measurement bias. In addition the same operator will perform the additional echocardiograms on the same patient for serial measurements. The median inter- and intra-observer variability has been shown to be 18 and 8.1 percent respectively (Kluckow, 2000).  Sample Size  We have recently completed our retrospective review of premature infants who had functional echocardiography in the first 48 hours of life. In our review infants who developed IVH or MRI abnormalities had a mean SVC flow of 83± 42  ml/kg/min vs 129 ± 67 ml/kg/min in those who did not. A sample size calculation determined that 23 infants in each group would be needed to show this difference, with a two-sided alpha of 0.05 and 80% power. Therefore, the sample size for the study will be 46 subjects; 23 infants randomized to the UCM arm and 23 to the ICC arm. Each patient will have 3 echocardiograms performed giving a total of 138 sample points in each of the described categories. The lowest SVC flow will be used as the comparison value. |
| **9. HUMAN SUBJECTS:** |
| This project will take place in the Infant Special Care Center (ISCC) at UCSD Medical Center in San Diego. The ISCC is a downtown, 49 bed neonatal unit with both Level II and Level III care, specializing in the care of high risk infants from birth until discharge to home. The subjects for this study will be infants < 32 weeks gestation randomly assigned to receive immediate cord clamping (<10 seconds) or umbilical cord milking (milking about 20 cm of umbilical cord three times over a total of 10 seconds to complete). Last year in 2010 the UCSD obstetric service delivered 110 infants < 32 weeks.  Exclusion criteria – obstetrician’s refusal to participate, surrogate delivery, parental desire for cord blood banking, major congenital anomalies, severe maternal illness, placental abruption or previa, if a ruptured uterus is found at delivery, hemoperitoneum. |
| **10. RECRUITMENT AND SCREENING:** |
| Potential patients will be identified by either the Research Coordinator, or by collaborators on the study or the PI by screening the labor and delivery service for eligible infants antenatally. |
| **11. INFORMED CONSENT:** |
| Parents will be approached prior to delivery. The principal investigator, Research Coordinator or other collaborators on the study will be obtaining consent. |
| **12. ALTERNATIVES TO STUDY PARTICIPATION:** |
| Our current practice in delivery of preterm neonates in immediate cord clamping. Echocardiography is part of routine care in our ICU for infants who are ill and may need medical therapy. |
| **13. POTENTIAL RISKS:** |
| Though echocardiography is a routine part care of ill neonates in the ICU. There are no known adverse effects of echocardiography. Echocardiography is a safe procedure performed routinely in the neonatal intensive care unit.  Groves et al (2005) showed that repeated echocardiography can be performed safely without any clinically significant changes in blood pressure, heart rate, or oxygen saturation. The patient may experience slight discomfort during the procedure. All efforts will be made to keep the infants as comfortable as possible such as swaddling and warming the ultrasound gel. Occasionally there will be the potential for desaturations, bradycardias, or tachycardias that occur with the extra handling that occurs with performing bedside ultrasound.  Umbilical cord milking has been performed in term infants for many years and is considered a safe practice. There have been small studies in preterm infants without adverse outcomes. There are no known adverse effects of umbilical cord milking but there may be risks that currently unforeseeable.  There is a risk of loss of patient confidentiality. In order to minimize the possibility of loss of confidentiality all patient data forms will be coded with a subject ID number and will not contain the name or medical record number. A single key to the coded subject ID numbers will be kept securely in a locked office. Echocardiograms will be stored with patient names and will be kept on DVD’s in a locked office. |
| **14. RISK MANAGEMENT PROCEDURES:** |
| During echocardiograms infants will be maintained in their current care environment and will be swaddled and  provided comfort measures such as pacifiers or feeds as appropriate for their clinical condition. No sedation will be  given specifically for the procedure. The infant will be monitored during the procedure by the physician, a pulse  oximeter and cardiorespiratory monitor the NICU. If the infant develops transient bradycardia during the procedure the echocardiogram will be paused until the heart rate recovers to baseline. If more than 2 episodes of bradycardia occur during any procedure the echocardiogram will be stopped completely. If at any time the echocardiographer (Dr. Leone or Dr. Katheria), the clinical attending physician or the bedside nurse caring for the infant at the time, feel that the infant is too unstable to tolerate the echocardiogram, it will not be performed. The parents may also decide at any time to withdraw the infant from the trial.  Patient protected health information will remain confidential. Each study patient will be assigned a study number which  will be used on all study data collection forms used for data analysis. The log with study numbers and patient  identifiers will be kept in the locked Investigative Drug Services office for security. Only the principal investigator and  co-investigators will have access to the data. Following completion of the study, the log and patient identifiers will be  destroyed. Though extreme care will be taken to keep all records confidential, there is a very small chance for loss of  confidentiality during the study period. |
| **15. POTENTIAL BENEFITS:** |
| The benefit to an individual patient involved in this trial is the potential for improved systemic blood flow if the patient is randomized to the umbilical cord milking arm. |
| **16. RISK/BENEFIT RATIO:** |
| There are unforeseeable risks in addition to echocardiograms and loss of patient data given that this intervention has been performed in two small trials. However, the risks are minor compared to the potential benefit in the interventional arm. |
| **17. EXPENSE TO SUBJECT:** |
| There is no expense to the subjects. Patients will not be billed for any of the exams. The complete blood counts (CBC) that are ordered for the study will be billed and paid for by the department of Neonatology. If the CBC is ordered for clinical purposes it will not be repeated for the study. Any other collected information is part of routine neonatal care and will be billed as such. |
| **18. COMPENSATION FOR PARTICIPATION:** |
| None. |
| **19. PRIVILEGES/CERTIFICATIONS/LICENSES AND ROLES OF THE RESEARCH TEAM:** |
| Anup Katheria is a physician with a California Medical License (A92926). He has Board Certification in General Pediatrics. He has privileges to practice at (PID#60599) at UCSD Medical Center.  Tina Leone is a physician with a California Medical License (A79693). She is Board Certified in Neonatal-Perinatal Medicine. She has privileges to practice (PID#50776) at UCSD Medical Center. - Co-Investigator  Doug Woelkers is a perinatologist with a California Medical License (G77134). He is Board Certificed in Maternal-Fetal Medicine. He has privileges to practice (PID#6923) at UCSD Medical Center. - Co-Investigator  Wade Rich, RRT, CCRC – Study Coordinator  Renee Bridge, RN – Study Nurse |
| **20. BIBLIOGRAPHY:** |
| Goldberg, R. N., D. Chung, et al. (1980). "The association of rapid volume expansion and intraventricular hemorrhage in the preterm infant." J Pediatr **96**(6): 1060-1063.  Groves, A. M., C. A. Kuschel, et al. (2005). "Cardiorespiratory stability during echocardiography in preterm  infants." Arch Dis Child **90**(1): 86-87.  Hosono, S., H. Mugishima, et al. (2008). "Umbilical cord milking reduces the need for red cell transfusions  and improves neonatal adaptation in infants born at less than 29 weeks' gestation: a randomised controlled  trial." Arch Dis Child Fetal Neonatal Ed **93**(1): F14-19.  Hosono, S., H. Mugishima, et al. (2009). "Blood pressure and urine output during the first 120 h of life in  infants born at less than 29 weeks' gestation related to umbilical cord milking." Arch Dis Child Fetal Neonatal  Ed **94**(5): F328-331.  Hunt, R. W., N. Evans, et al. (2004). "Low superior vena cava flow and neurodevelopment at 3 years in very preterm infants." J Pediatr **145**(5): 588-592.  Kluckow, M. and N. Evans (2000). "Low superior vena cava flow and intraventricular haemorrhage in preterm infants." Arch Dis Child Fetal Neonatal Ed **82**(3): F188-194.  Mc, C. A., F. Holmes, et al. (1949). "Management of cord and placental blood and its effect upon the  newborn." Calif Med **71**(3): 190-196.  Mc, C. A., F. Holmes, et al. (1950). "Management of cord and placental blood and its effect upon the  newborn. Part II." West J Surg Obstet Gynecol **58**(11): 591-608.  Mercer, J. S., B. R. Vohr, et al. (2006). "Delayed cord clamping in very preterm infants reduces the incidence of intraventricular hemorrhage and late-onset sepsis: a randomized, controlled trial." Pediatrics **117**(4): 1235-1242.  Rabe, H., G. Reynolds, et al. (2008). "A systematic review and meta-analysis of a brief delay in clamping the umbilical cord of preterm infants." Neonatology **93**(2): 138-144.  Skinner, J. R., R. J. Boys, et al. (1991). "Non-invasive assessment of pulmonary arterial pressure in healthy neonates." Arch Dis Child **66**(4 Spec No): 386-390.  Rabe, H., Jewison A, et al (2011). “Milking compared with delayed cord clamping to increase placental transfusion in Preterm neonates: A Randomized Controlled Trial. Obstet Gynecol. 117:205-211. |
| **21. INDUSTRY-SPONSORED OR COLLABORATING STUDIES:** |
| Not Applicable |
| **22. OTHER FUNDING SUPPORT FOR THIS STUDY:** |
| UCSD department of Neonatal-Perinatal Medicine |
| **23. BIOLOGICAL MATERIALS TRANSFER AGREEMENT:** |
| Not applicable |
| **24. INVESTIGATIONAL DRUG FACT SHEET:** |
| Not applicable |
| **25. IMPACT ON STAFF:** |
| The bedside nurses caring for the study patients will be informed of the need for each study. They will not be required to assist in the measurements but will notify the operator of any instability of the infant during the study. |
| **26. CONFLICT OF INTEREST (COI)** |
| The principal investigator has no conflict of interest. |
| **27. SUPPLEMENTAL INSTRUCTIONS FOR CANCER-RELATED STUDIES** |
| Not applicable |
| **28. PROCEDURES FOR SURROGATE CONSENT AND/OR DECISIONAL CAPACITY ASSESSMENT** |
| Surrogates will not be approached for consent in our study. |
